# Male Victims of Domestic Violence: Clinical and Behavioral Insights from an Italian Hospital-Based Study

**DOI:** 10.3390/bs16030353

**Published:** 2026-03-02

**Authors:** Martina Focardi, Paola D’Onofrio, Monique Cestaro, Marta Guerini, Francesca Romana Ermini, Marco Carnevali, Rossella Grifoni, Barbara Gualco, Ilenia Bianchi, Vilma Pinchi, Beatrice Defraia

**Affiliations:** 1 Forensic Pathology Unit, AOU Careggi, Largo Brambilla 3, 50134 Florence, Italy; martina.focardi@unifi.it (M.F.); monique.cestaro@unifi.it (M.C.); marta.guerini@unifi.it (M.G.); rossella.grifoni@unifi.it (R.G.); beatrice.defraia@unifi.it (B.D.); 2 Rosa Code Unit, AOUC-Careggi University Hospital, 50134 Florence, Italy; carnevalim@aou-careggi.toscana.it; 3 Hospital Management Rosa Code Unit, AOUC-Careggi University Hospital, 50134 Florence, Italy; donofriop@aou-careggi.toscana.it; 4 Santa Maria Nuova Hospital, 50122 Florence, Italy; erminifr@aou-careggi.toscana.it; 5 Forensic Medical Sciences, Department of Health Science, University of Florence, Largo Brambilla 3, 50134 Florence, Italy; barbara.gualco@unifi.it; 6 Laboratory of Personal Identification and Forensic Morphology, Department of Health Sciences, University of Florence, Largo Brambilla 3, 50134 Florence, Italy; vilma.pinchi@unifi.it

**Keywords:** intimate partner violence, male victims, help-seeking behavior, domestic violence, gender norms, behavioral barriers, forensic assessment, emergency medicine, victimization patterns

## Abstract

Domestic violence against men remains significantly under-recognized, despite affecting 20–40% of men worldwide. Societal stigma, gender-normative expectations, and institutional biases often discourage help-seeking behaviors among male victims. This retrospective analysis characterizes domestic violence against adult men by examining victim–perpetrator dynamics, injury patterns, reporting behaviors, and behavioral barriers to help-seeking within an Italian emergency department setting. Overall, 80 adult male domestic violence victims presenting to the Emergency Department of Careggi University Hospital (Florence, Italy) between January 2017 and December 2022 were examined. Data included demographics, injury characteristics, perpetrator relationships, and formal reporting rates. Descriptive statistics and chi-square tests were used to examine associations between victim characteristics and help-seeking behaviors. The majority of victims were Italian men (age range 18–90 years, mean 44.2 ± 15.1); of these, 55% experienced IPV perpetrated by female partners. Physical injuries were predominantly minor (classified as minor according to ED prognosis ≤ 7 days) (78.8%), including abrasions and contusions affecting the head (52.5%), neck (28.8%), and upper limbs (41.3%). Formal reports were filed with judicial authorities in 58.8% of cases, yet only 15% accepted protective interventions. Visible facial injuries (OR = 3.85, 95% CI, *p* = 0.004) and female perpetrators (OR = 8.23, 95% CI, *p* < 0.001) were independent predictors of formal reporting. Documented behavioral barriers included stigma (68%), fear of disbelief (45%), and adherence to traditional masculine norms (52%). Our findings demonstrate that male domestic violence victims face substantial behavioral and systemic barriers that prevent help-seeking. Enhanced clinical–forensic training, gender-inclusive response protocols, and public awareness campaigns are essential to provide equitable support and reduce under-reporting.

## 1. Introduction

Domestic violence (DV) represents a pervasive public health and societal concern that encompasses a broad spectrum of abuse—physical, emotional, psychological, sexual, and economic—typically occurring within intimate or familial relationships. Over recent decades, public discourse, research, and institutional responses have largely focused on women as victims and men as perpetrators, thereby shaping policies, clinical protocols, and social expectations accordingly (World Health Organization, 2002; Kolbe & Büttner, 2020; Campbell, 2002).

While this gendered perspective has been essential for addressing violence against women, it has inadvertently marginalized male victims of domestic violence (Hines & Douglas, 2010).

Emerging research, however, has begun to challenge the assumption that men are predominantly aggressors, demonstrating that a significant minority of men also experience abuse—often in silence. Studies across Europe, the United States, and Australia suggest that 20–40% of men report experiencing some form of intimate partner violence (IPV) in their lifetime (Dobash & Dobash, 2004; Archer, 2000; Deli et al., 2025; Australian Bureau of Statistics, 2022; Office for National Statistics (UK), 2023). Within the research literature, IPV is often used interchangeably with domestic violence (DV), though important distinctions exist. The IPV has a narrower definition than DV (Basile et al., 2025). Specifically, DV is defined as violence occurring within familial or household settings, encompassing a broad range of relationships, while IPV refers to harm inflicted by current or former intimate partners, including physical, sexual, psychological, and economic abuse (Basile et al., 2025). Officially, the World Health Organization—WHO defines IPV as a “behavior within an intimate relationship that causes physical, sexual, or psychological harm, including acts of physical aggression, sexual coercion, psychological abuse, and controlling behaviors” (World Health Organization, 2012).

Despite these prevalence estimates, male victimization continues to be under-reported and poorly understood. Societal stigma, rigid gender norms, and institutional biases often prevent men from recognizing abuse, disclosing abuse, or seeking help (Swan et al., 2008; Mechem et al., 1999).

The psychological and sociocultural factors that shape male victimization are complex and have been often neglected in both clinical settings and research. Ideals of masculinity, combined with fear of stigmatization and anticipated disbelief can lead to significant under-reporting and, consequently, under-recognition of male victims. Moreover, institutional responses—such as protocols primarily designed for female victims—may inadvertently marginalize men. Understanding these behavioral barriers is crucial not only for clinical diagnosis and forensic evaluation but also for designing inclusive support systems and public health interventions (Dutton & Nicholls, 2005; Centers for Disease Control and Prevention [CDC], 2024). When unaddressed, these barriers result in delayed or denied access to protection, justice, and care.

Within clinical and emergency settings, protocols and training programs often fail to consider male victims as a distinct population, thereby leading to challenges in detection, documentation, and intervention. Forensic and healthcare professionals may inadvertently overlook signs of abuse due to cognitive biases or lack of experience with male victims. Consequently, many men do not receive appropriate support, and their experiences remain invisible within public health and legal systems (Harvey et al., 2007).

In recent years, however, the scientific community has begun to examine male-directed violence more closely, aiming to identify predictive factors, assess consequences, and develop effective support strategies (Cook, 2009; Morgan & Wells, 2016; Council of Europe Convention, 2011; Thomas & Kopel, 2023). Several behavioral frameworks help explain the persistent under-reporting among male domestic violence victims (Lysova et al., 2020).

Social cognitive theory posits that help-seeking behavior is influenced by self-efficacy beliefs, outcome expectations, and observational learning from social models. Because male victims frequently lack positive role models of men seeking help for violence, this absence reduces their perceived self-efficacy for disclosure.

Similarly, the Health Belief Model suggests that help-seeking behavior depends on perceived severity, susceptibility, benefits, and barriers. Male victims of domestic violence, and especially male victims of IPV, may downplay perceived severity due to socialization emphasizing physical toughness, while perceived barriers (stigma, legal discrimination, lack of services) far outweigh perceived benefits (Peraica et al., 2021).

Additionally, gender role conflict theory explains how adherence to restrictive masculine norms creates psychological distress when men experience victimization, leading to avoidance coping strategies rather than help-seeking. Understanding these behavioral mechanisms is essential for developing effective, gender-sensitive interventions (Sita & Dear, 2021).

Recent systematic reviews have identified gender bias, personal fears, and familial factors as primary barriers to male help-seeking, and have recommended multi-faceted interventions. Cultural stigma, fear of disclosure, and negative institutional responses consistently drive men’s reluctance to seek formal help across diverse cultural contexts. Among Black men, intersecting barriers shaped by hegemonic masculinity and structural racism particularly complicate recognition of victimization and encourage informal over formal help-seeking (Stewart & Haselschwerdt, 2023, 2025; Kim et al., 2024; McLeod et al., 2024; Lane et al., 2023; Landa-Blanco & Mejía Sánchez, 2025).

Against this background, the present study addresses this gap by offering a retrospective analysis of male victims of domestic violence who presented at the Emergency Department (ED) of Careggi University Hospital in Florence, Italy, between 2017 and 2022, with particular focus on victim–perpetrator dynamics, injury patterns, reporting rates, and the behavioral responses of male victims—particularly their acceptance or refusal of protective services. Our research aims to highlight critical shortcomings in current practices, thereby supporting calls for more inclusive, gender-sensitive approaches to domestic violence. This study contributes to a growing body of literature urging a shift in public health and forensic paradigms to acknowledge and respond to male victimization with the same urgency afforded to other forms of domestic abuse. Ultimately, understanding how male victims experience, internalize, and respond to violence is essential for developing effective interventions that respect the complexity of gender, vulnerability, and trauma.

## 2. Materials and Methods

### 2.1. Study Design and Setting

This investigation aims to investigate the clinical, demographic, and behavioral characteristics of adult male victims of domestic violence who accessed emergency medical care over a six-year period, from 1 January 2017 to 31 December 2022. We addressed the following research questions:

1. What are the demographic and clinical characteristics of male victims of domestic violence presenting to emergency services?
2. What injury patterns distinguish male domestic violence victimization in cases of IPV or non-IPV?
3. What behavioral and psychological barriers prevent male victims from reporting abuse and accepting help?
4. How do perpetrator characteristics relate to victim help-seeking behaviors?

A retrospective, monocentric study was conducted at the ED of Careggi University Hospital in Florence, Italy, which serves as the primary regional facility for acute care and has implemented the Rosa Code protocol—an interdisciplinary response pathway for victims of interpersonal violence, including DV and IPV.

The Rosa Code protocol provides the best social and health support for victims of any form of violence, male or female, who seek help from the emergency services in Tuscany. Regional social services complement this protocol to ensure a complete assistance pathway. Originally created to assist victims of female violence and victims of hate crimes, the network can also be accessed by male domestic violence victims and other vulnerable people (Krug et al., 2002; Houseman et al., 2024; Wörmann et al., 2021; Focardi et al., 2022, 2024a, 2024b).

When a patient, regardless of gender, enters the ED alleging an episode of domestic violence, or when there is a suspicion of violence (defined as an incident that is not declared but suspected based on risk factors, suspicion indicators, and clinical judgment by healthcare professionals), they are accompanied by the healthcare staff to a specific exam room, where their physical and psychological needs are attended to by medical staff specifically trained in treating victims of violence.

Since 2018, in instances of declared or suspected violence, healthcare workers have been required to fill out a specific form as part of the Rosa Code protocol, in addition to standard medical reports. This form records a detailed description of the violence and accurate information about the injuries, such as location, detailed descriptions, and severity. Photographs are also included. The form also records past episodes and the possible risk of recurring violence. Furthermore, an evaluation is made regarding whether to inform the authorities and to assess if returning home is an option or if local sheltering and safety protection is necessary. Healthcare workers also proceed with sampling biological material for toxicological or genetic forensic investigations, which are conducted via collecting samples/swabs according to the patients’ dynamics or needs (Associazione Scientifica Gruppo Tossicologi Forensi Italiani [GTFI], 2017).

The Rosa Code network, which involves all professionals assisting victims of violence (physicians, nurses, social workers, law enforcement officers, anti-violence centers, etc.), represents a model based on national ministerial guidelines that can be effectively transferred to other settings, both with regard to the healthcare and social management of the victim and also to the forensic pathway.

It is important to note that domestic violence research and intervention efforts have predominantly focused on women as victims, which, while crucial, has led to a gendered blind spot in policies and care systems (World Health Organization, 2002; Kolbe & Büttner, 2020; Campbell, 2002; Harvey et al., 2007).

### 2.2. Data Collection

This study was conducted in accordance with the Declaration of Helsinki and approved by the Ethics Committee of Careggi University Hospital (Protocol Code: Approval No. 20160118).

All data were stored securely in password-protected databases accessible only to authorized research personnel. Patient identifiers were removed and replaced with unique study codes. The research team followed strict protocols to maintain confidentiality throughout data collection, analysis, and reporting.

Participants were included if they were male, aged 18 years or older, and identified as declared or suspected victims of domestic violence at the time of ED evaluation.

We excluded female patients, individuals under 18 years of age, and cases involving non-domestic violence (e.g., hate crimes, sexual assault, accidental injuries).

Minors were excluded, as pediatric victims of violence are referred to specialized children’s hospitals.

Data were extracted from medical records and Rosa Code forms compiled by emergency personnel at the time of admission. For each case, the following information was collected:

- Demographic variables (age, nationality, etc.);
- Access details (time of ED arrival, self-presented or via ambulance arrival, triage code assigned, etc.);
- Temporal information (time elapsed between the violent episode and hospital admission);
- Circumstantial data (setting of violent incident, relationship to the perpetrator, number of aggressors, etc.);
- Clinical data: type of violence (in instances of blunt trauma violence, we distinguish between instrumental violence and non-instrumental violence, e.g., punches/kicks), number of events, and anatomical location of injuries, medical diagnoses, prognosis, and whether hospitalization was required;
- Reporting and legal response (whether the case was reported to law enforcement or judicial authorities);
- Protective services, in particular whether territorial social support was offered and accepted: when support was declined, we documented the reason for refusal, categorized into seven response categories.

### 2.3. Statistical Analysis

Descriptive statistics were calculated for all variables. Continuous variables were expressed as mean ± standard deviation (SD) or median with interquartile range (IQR) depending on distribution normality (assessed using Shapiro–Wilk test). Categorical variables were expressed as frequencies and percentages.

- Chi-square tests or Fisher’s exact tests (when expected cell counts < 5) were used to compare categorical variables between groups (e.g., reporters vs. non-reporters, help-acceptors vs. refusers).
- Independent *t*-tests or Mann–Whitney U tests were applied for continuous variables depending on distribution normality.
- Logistic regression models were constructed to identify predictors of reporting behavior and help-seeking acceptance, adjusting for age, nationality, and injury severity as potential confounders.

A two-tailed *p*-value < 0.05 was considered statistically significant. All analyses were performed using SPSS version 31.

Cases with missing data on key variables were excluded from relevant analyses.

## 3. Results

Between 2017 and 2022, 80 adult male victims of domestic violence were identified out of a total of 1071 cases (991 women and 80 men). The patients ranged in age from 18 to 90 years, with a mean age of 48 years. The majority were Italian nationals (83.8%), followed by African individuals (7.5%), European Union citizens (5%), South American individuals (2.5%), and Asian individuals (1.2%).

The annual case distribution revealed the highest incidence in 2017 (31.2%) and 2022 (20%), with a marked decline in 2020 (7.5%) and 2021 (10%), potentially influenced by the COVID-19 pandemic (Table 1).

According to the age distribution, most cases occurred among individuals aged 41–60 years (53.8%), followed by the 18–30 age group (22.5%). Only 7.5% of victims were over 70 years old (Table 1).

ED visits occurred most frequently in the afternoon and evening hours (46.2%), followed by nighttime (29.4%) and morning (24.4%). Most patients self-presented (83.8%), while 15% arrived via ambulance, indicating that the majority perceived the situation as manageable without emergency transport (Table 2).

The time between the violent incident and hospital presentation varied: overall, 42.5% accessed care within two hours, with an average delay of approximately 8 h. However, 30% of cases lacked clear documentation of timing.

Details regarding the characteristics of perpetrators and relationships are presented in Table 2.

In 63% of cases, the aggressor was female (50/80). In 55% of cases (44/80), violence occurred within heterosexual intimate partner relationships (IPV). Same-sex IPV was recorded in only two cases. In 88.8% of cases, the violence occurred in a domestic setting, reinforcing the private, often concealed nature of abuse.

The study disclosed a distinct injury profile among male victims. Physical violence was documented in all cases, with the majority (78.8%) involving direct physical contact such as punches, kicks, slapping, or pushing (Table 3). Analysis revealed that the most common injury types included abrasions (30%), contusions and hematomas (21.3%), combined injuries (abrasions associated with contusions or injuries involving functional impairment or bone fractures), and bite marks, which were observed in seven cases (8.8%) (Table 3). Injuries typically involved the head, neck, and upper limbs, aligning with known IPV injury patterns. Multiple anatomical regions were affected in 43.75% of cases, suggesting repeated or sustained physical aggression (Table 3).

Notably, all bite marks occurred in IPV contexts, predominantly affecting the upper limbs (four cases) and torso (three cases).

In cases of blunt-force trauma, 17% were due to instrumental injury, while 83% resulted from non-instrumental violence (fists and kicks). Only one patient required hospitalization. The majority (62.5%) received a prognosis of ≤7 days, with an additional 17.5% receiving a prognosis of between 8 and 15 days. Extended prognoses were less common: one case received 20 days (1.25%), two cases received 30 days (2.5%), while two cases had no established prognosis, and prognosis data were unavailable in 12.5% of cases.

Regarding lesion severity, the assigned triage severity code (a color-coded system used in Italy) was green in 79% of cases and yellow in 21% of cases.

Out of the total sample (80 cases), formal reports to judicial authorities (public prosecutor or police) were filed for 47 subjects (58.8%), following standard legal requirements for suspected domestic violence. Notably, 34.7% of victims disclosed prior experiences of abuse that had not been formally reported. Table 4 presents a comparative analysis of characteristics between reporters and non-reporters.

As shown in Table 4, the 47 male victims (58.8%) who filed formal reports exhibited several recurring patterns. Compared to non-reporters, they were significantly more likely to present with visible facial injuries (68.1% vs. 35.3%, χ^2^ = 8.64, *p* = 0.003), multiple injury sites (mean 2.8 ± 1.2 vs. 1.6 ± 0.8, t = 5.23, *p* = 0.001), female perpetrators (87.2% vs. 45.5%, χ^2^ = 15.42, *p* < 0.001), and previous ED visits for DV (34.0% vs. 12.1%, χ^2^ = 5.18, *p* = 0.012). Multivariate logistic regression analysis revealed that visible facial injuries (OR = 3.85, 95% CI: 1.52–9.74, *p* = 0.004) and female perpetrators (OR = 8.23, 95% CI: 2.91–23.3, *p* < 0.001) were independent predictors of formal reporting after adjusting for age, nationality, and injury severity.

Among victims who did not file formal reports (n = 33), medical record documentation revealed multiple, often overlapping, barriers to help-seeking and reporting, as detailed in Table 5. The documented information reflects statements provided by victims to examining physicians during medical evaluation, categorized into seven distinct themes. Some victims reported multiple concurrent reasons for non-reporting. Stigma and shame emerged as the most prevalent barrier, following the adherence to traditional masculine norms and the fear of not being believed, in particular by the authorities or social services. A substantial proportion of non-reporters victims engaged in fearing a retaliation of perpetrators, in particular in cases involving minors custody or parental rights.

The minimization of abuse, downplaying the severity of the violence or rationalizing it as an isolated incident, resulted to represent he most common adopted coping strategy among male victims (Table 5).

Regarding the activation of protective services, healthcare staff proposed territorial interventions (e.g., shelters, social service follow-up) in 47.5% of cases (38 cases). However, uptake was markedly low: only 12 cases (31.6%) accepted support, while 26 (68.4%) explicitly refused. Acceptance patterns varied significantly by nationality. All victims who accepted services were Italian nationals (100%), whereas all South American patients and half of African patients declined assistance, suggesting potential cultural barriers or distrust of formal support systems.

## 4. Discussion

This retrospective study contributes to the limited but growing body of evidence documenting male victimization in domestic violence contexts. The authors sought to characterize key patterns in injury profiles, perpetrator relationships, and help-seeking behaviors among male victims. Our findings carry significant implications for both public health policy and clinical practice, particularly regarding the recognition, assessment, and support of an underserved victim population.

Domestic violence research and intervention have historically focused predominantly on female victims and male perpetrators, a pattern that reflects both the epidemiological reality that women constitute the majority of severe domestic violence victims and the gendered nature of intimate partner violence (Houseman et al., 2024). This focus has driven the establishment of comprehensive support systems worldwide, including in Italy, encompassing specialized healthcare services, legal protections, emergency shelters, and educational programs designed specifically for female victims. Nevertheless, male victimization in domestic violence contexts remains substantially underestimated and under-researched. Social and psychological barriers—including stigma, gender role expectations, and fear of disbelief—contribute to persistent underreporting and result in fragmented data in the scientific literature (Harvey et al., 2007; Wörmann et al., 2021). Consequently, despite increasing general awareness of domestic violence as a public health issue, male victims continue to be marginalized in both public discourse and institutional responses. This marginalization is reflected in the scarcity of male-specific assessment tools, limited availability of appropriate support services, and inadequate training of healthcare professionals to recognize and respond to male victimization.

We collected data on male domestic violence victimization from the ED of the Careggi University Hospital, the main tertiary hospital in Florence, Italy. Data were obtained from clinical reports of domestic violence perpetrated against men accessed to the Rosa Code protocol from 1 January 2017 to 31 December 2022.

From a total of 1071 cases of domestic violence victims, 80 were men and 991 were women. A similar proportion was found by Thureau et al. (2015), although other European studies have documented varying percentages, likely reflecting differences in healthcare systems, reporting practices, and cultural contexts (Kolbe & Büttner, 2020).

Comprehensive national data on domestic violence against men in Italy are not currently available, thus precluding direct comparison of our findings with national trends. The solely interesting information on violence perpetrated against man within a domestic environment can be assumed from an ISTAT survey (2018) conducted in Italy over the 2015–2016 period. The registered data disclosed that 3,754,000 men (corresponding to 18.8% of the total male population) have experienced sexual abuse during their lifetime. It should also be noted that 435,000 men, equal to 2.2%, were victims of sexual abuse before the age of 18. Importantly, the ISTAT data indicated that perpetrators of sexual abuse against men were predominantly male, contrasting with our findings regarding domestic violence, where female perpetrators predominated. This discrepancy underscores the importance of distinguishing between sexual abuse and other forms of domestic violence, as perpetrator gender patterns, contextual factors, and victim responses may differ substantially across violence types.

Previous Italian research by Margherita et al. (2021) analyzed a sample of 119 men victims of domestic violence from 2014 to 2018 and observed a steadily increasing trend in reported cases over time. Our sample revealed a relatively stable annual case distribution across the study period, with one notable exception: a marked decline during 2020 and 2021, corresponding to the COVID-19 pandemic during which a general limited access to Health Services was registered. This reduction likely reflects restricted access to healthcare services during lockdown periods rather than a genuine decrease in violence incidence. Indeed, national data from the same period documented increased calls to emergency domestic violence hotlines and overall increases in reported domestic violence incidents (Osservatorio Sociale Regionale, 2023), suggesting that the pandemic may have simultaneously increased violence while creating barriers to formal healthcare access. This trend has been reported in other countries (Boserup et al., 2020) highlighting the vulnerability of male victims during public health crises when informal help-seeking pathways may be disrupted.

According to the age distribution, only 7.5% of victims were over 70 years old with a prevalence of individuals aged between 41–60 years (53.8%) and 18–30 years (22.5%). The finding suggests that middle-aged men are more likely to report or seek treatment for abuse, but also a possible underreporting among elderly males. The underrepresentation of elderly male victims warrants particular attention from both clinical and public health perspectives, as this population may face unique vulnerabilities and barriers to help-seeking.

Regarding nationality, the majority of victims in our study are Italian, so the possible disadvantages or difficulties in the integration process of immigrants do not appear to be a risk factor in men experiencing domestic violence. Our limited sample size precludes definitive conclusions, but the underrepresentation of immigrant men may result from multiple intersecting barriers, including language difficulties that impede communication with healthcare providers, cultural factors that intensify stigma around male victimization, concerns about immigration status and interaction with authorities, distrust of institutions based on prior experiences in countries of origin, or reliance on informal community-based support networks rather than formal healthcare systems.

The temporal distribution of emergency department presentations revealed that the majority of visits occurred during afternoon and evening hours (46.2%), followed by nighttime (29.4%) and morning (24.4%). This pattern has important implications for healthcare resource allocation and staff training, suggesting that specialized personnel with expertise in recognizing and responding to male victimization should be prioritized during afternoon and evening shifts when presentation rates peak.

Time between violence and ED access as been identified as a prognostic and predictive factor of violence in many categories of victims, especially children and women (Thureau et al., 2015; Kos & Shwayder, 2006). However, our findings suggest that this relationship may differ for male victims since, in our sample, 42.5% of men presented within eight hours. The substantial proportion of cases with missing timing data highlights a documentation gap that should be addressed in clinical protocols, but the relatively prompt presentation among those with documented timing may reflect the fact that physical violence with physical injuries needing treatment was documented in all cases.

The perpetrators were registered as females in 62.5% of cases. This finding appears in partially contrast with the ISTAT (2018) national data indicating predominantly male perpetrators of violence against men. However, this apparent discrepancy reflects the distinction between sexual abuse (the focus of the ISTAT survey) and the broader category of domestic violence examined in our study. The predominance of female perpetrators in domestic violence contexts, particularly intimate partner violence, represents a well-documented pattern in male victimization research and underscores the importance of disaggregating violence types when interpreting epidemiological data. In fact, IPV accounted for 55.7% of cases, with female partners representing the vast majority of perpetrators in these incidents. This finding is consistent with previous European forensic studies (Houseman et al., 2024; Wörmann et al., 2021; Carmo et al., 2011; Savall et al., 2017; Straus, 2011). Male partners were perpetrators in only two cases, in agreement with most studies on IPV in males (Focardi et al., 2024b). In cases involving multiple perpetrators, family members other than intimate partners were typically implicated (Focardi et al., 2024b; Thureau et al., 2015), suggesting that domestic violence against men could include broader family conflict dynamics.

The low proportion of same-sex IPV and non-intimate perpetrators in our sample suggests that heterosexual IPV dynamics are most often represented, reflecting patterns of disclosure and healthcare system recognition rather than true prevalence of same-sex intimate partner violence in the population. In fact, scientific research (Rollè et al., 2018) emphasizes that violence occurring within the LGBTQ+ community is particularly underestimated and often not recognized by healthcare professionals, with significant consequences for the physical and psychological health of the victims.

Furthermore, only one-third of male victims reported previous episodes of domestic violence, all occurring in intimate partner contexts with a substantially lower rate than typically observed among female intimate partner violence survivors (Kim et al., 2024). This apparent lower recurrence rate requires careful interpretation, as it may reflect genuine differences in violence patterns, underreporting driven by male-specific barriers, or methodological limitations in data collection. Psychological barriers including internalized stigma, fear of disbelief, and normalization of violence, particularly when perpetrated by female partners, likely suppress disclosure of prior victimization (Swan et al., 2008; Mechem et al., 1999). Additionally, our data collection methodology may have contributed to underreporting since physicians completing standardized forms focused primarily on physical injuries when documenting “repeated episodes of violence,” potentially overlooking or not systematically inquiring about psychological, economic, emotional, or other non-physical forms of abuse that often precede or accompany physical violence. This methodological limitation underscores the need for comprehensive, validated assessment instruments that capture the full spectrum of domestic violence experiences rather than focusing exclusively on physical injury. Our findings contribute to the ongoing scholarly discourse regarding gender patterns in intimate partner violence perpetration and victimization. Straus (2011) introduced the concept of “gender symmetry” in intimate partner violence, documenting that roughly equal proportions of women and men perpetrate physical aggression against intimate partners in population-based studies. However, this symmetry in perpetration rates coexists with a “gender asymmetry” in consequences, with women typically suffering more severe physical injuries and psychological harm. Straus distinguished between “ordinary” violence, including slapping, shoving, and throwing objects, which is relatively common in the general population and demonstrates gender symmetry, and “severe” violence, including choking, punching, and weapon use, which is more prevalent in clinical populations and remains predominantly male-perpetrated. This theoretical framework has important implications for intervention design. Prevention programs targeting perpetration should address both male and female aggression, recognizing that intimate partner violence often involves bidirectional conflict and that early intervention with both genders may prevent escalation. Conversely, treatment programs must remain cognizant of gender asymmetries in injury severity and psychological impact, ensuring that male victims receive validation and appropriate support while not minimizing the disproportionate burden of severe intimate partner violence borne by female victims. Our data, showing predominantly female perpetration but generally minor, not severe physical injuries, align with this nuanced understanding of gender patterns in intimate partner violence (Straus, 2011).

The injury patterns documented in our sample of male victims were largely consistent with established DV research. Most cases involved blunt-force trauma, including abrasions and contusions, to the head, neck, and upper limbs—areas known to be commonly targeted in close-quarters aggression (Sheridan & Nash, 2007; Tam et al., 2010). While most injuries were classified as medically minor (79% received green triage codes indicating low acuity), their non-specific nature poses significant challenges for forensic interpretation. The substantial overlap between domestic violence injuries and those resulting from accidental trauma or self-inflicted harm necessitates comprehensive clinical assessment that integrates physical findings with behavioral observations, consistency of patient narrative, and contextual risk factors (Stewart & Haselschwerdt, 2023; Straus, 2011). The affect-loaded and situational nature of DV in our sample is reflected by the diagnosis of non-instrumental blunt violence in the majority of the analyzed cases, which is consistent with previous studies indicating that most domestic violence episodes involve impulsive, emotionally charged confrontations rather than premeditated, instrumental aggression (Focardi et al., 2024b; Thureau et al., 2015; Boserup et al., 2020; Carmo et al., 2011; Douglas & Hines, 2011). In fact, in the 17% of cases involving instrumental violence, perpetrators typically employed readily available household objects as weapons (Harvey et al., 2007). Lesions were frequently inflicted on the head, neck, upper limbs, and thorax, primarily causing abrasions and contusions/bruises. These findings are consistent with data collected from other studies that analyzed physical IPV (Straus, 2011; Centers for Disease Control and Prevention [CDC], 2024; Margherita et al., 2021). This pattern likely reflects both the accessibility of upper body regions during confrontations and potential defensive posturing by victims attempting to protect themselves. As stressed by Margherita et al. (2021), common injury types inflicted in the domestic setting, such as bruises and contusions to the head, neck, and upper body, can result from multiple etiologies including accidental trauma, self-inflicted harm, or interpersonal violence. This diagnostic complexity underscores the critical importance of clinician training in recognizing subtle indicators of domestic violence beyond obvious physical injuries. Forensic experts play a crucial role in the differential diagnosis of domestic violence and accidental or self-inflicted lesions, requiring sophisticated integration of multiple data sources, accurate assessment of the physical examination findings, evaluation of injury pattern consistency with the reported mechanism, investigation of psychological and behavioral risk factors, consideration of the patient’s narrative and disclosure patterns, and combination of objective clinical findings with contextual information about the patient’s social circumstances and relationship dynamics.

Of particular interest are the seven cases of bite marks, all occurring in IPV contexts when perpetrator were female partners. Although rare in this sample, bite wounds are forensically significant due to their intimate nature and potential for identification through dental analysis (Allen-Collinson, 2009; Archer, 2000). Their exclusive occurrence in IPV cases underlines the emotional and impulsive dimensions of such assaults considering that all the registered bite mark injuries were, however, superficial and never severe. While bite marks were relatively uncommon in Italy, international research documents higher prevalence in IPV cases in other countries, particularly when perpetrators are female (Savall et al., 2017).

Most patients received low-acuity triage codes (79% green code) and did not require hospitalization (only one case), which aligns with the perceived “mildness” of physical injuries. However, while these findings might superficially suggest relatively “mild” trauma, such characterization risks substantially minimizing the broader health consequences of victimization. The apparent mildness of acute physical injuries does not correlate with psychological impact, particularly when abuse is recurrent, occurs within intimate relationships, or is accompanied by emotional manipulation, economic control, and social isolation (Table 5). This disconnect between physical injury severity and overall health impact is especially understandable for male victims, who face additional burdens of societal invalidation, gender role conflict, and severely limited access to gender-appropriate support services. Healthcare providers must therefore avoid equating low physical injury severity with low clinical significance and should maintain vigilance for psychological sequelae even when physical injuries appear minor.

One of the most striking findings of this study is the markedly low acceptance rate of protective measures despite clear evidence of domestic abuse. Only 15% of victims agreed to engage with territorial support services, while one-third declined, and nearly half were not deemed eligible for intervention. The reluctance to accept help likely reflects a complex interplay of shame, denial, fear of social judgment, and the internalized belief that men must be self-reliant. Documented barriers in our sample included shame and embarrassment about victimization (66.7% of non-reporters), fear of disbelief by authorities (45.5%), fear of perpetrator retaliation (33.3%), desire to protect children from family disruption (24.2%), and belief that violence would cease without intervention (21.2%). These findings align with established behavioral theories linking gender role expectations with underreporting and help avoidance among male victims (Dutton & Nicholls, 2005; Morgan & Wells, 2016; Hines & Malley-Morrison, 2001), while also highlighting the specific manifestation of these barriers in the Italian cultural context.

Although the small sample size does not allow for general considerations, sociocultural background may further influence behavior. Notably, 100% of South American men and 50% of African men in the sample declined protective services, compared to 33% of Italians. This could suggest that cultural stigma, distrust of institutions, or language barriers may be stronger in certain populations. Tailored outreach, culturally sensitive support models, and better interpreter access may be critical in addressing these disparities and has to be recognized by the healthcare systems.

Despite the implementation of the *Rosa Code* protocol to include different victims of violence, current tools remain female-centric. In fact, the risk assessment questionnaire administered during ED admission includes only four questions applicable to male victims, severely constraining comprehensive evaluation of danger levels and appropriate tailoring of support services. This gender imbalance in assessment tools reflects broader systemic assumptions about domestic violence that may impede recognition and appropriate response when victims are male. Additionally, clinical documentation frequently failed to address whether minor children were present during violent incidents or exposed to ongoing domestic conflict against men, a critical legal consideration under Italian Law 69/2019 and an essential factor for assessing family-level risk and child welfare needs (Osservatorio Sociale Regionale, 2023). This documentation gap potentially reflects implicit assumptions that male victims are less likely to be primary caregivers or that children’s exposure to maternal violence is less concerning than exposure to paternal violence, biases that require explicit attention in clinical training and protocol development. Thus these findings highlight the need to revise protocols and guidelines to include male-specific indicators, risk factors, and outcomes, and train ED staff for gender-sensitive approaches to interviewing, documenting, and interpreting signs of violence in male patients. Standardized documentation should include questions about children’s presence and exposure to violence regardless of victim gender, eliminating differential documentation practices based on parental gender.

Legal reporting to judicial authorities (prosecutor or police) occurred in 58.8% of cases, as mandated by Italian law for suspected or declared domestic violence. However, documentation quality and comprehensiveness varied considerably. Only half of reported cases included information about previous violent episodes, potentially reflecting physicians’ tendency to focus primarily on acute physical injuries while overlooking or not systematically inquiring about prior psychological, economic, verbal, or other non-physical abuse that often precedes or accompanies physical violence. This documentation gap limits understanding of violence patterns, escalation trajectories, and cumulative risk. A further cause for concern is that neither in this case, and confirming the bias revealed from the ED questionnaire formulation, children’s presence during violent episodes was registered. Not only the idea of the man as a victim, but more over of the father as a victim of female violence, is not yet fully accepted and it is not deeply understood that violence is violence and that minors are victims of both the violence experienced by the mother and that perpetrated by the father (Douglas & Hines, 2011; Focardi et al., 2022).

Our findings align with established behavioral theories explaining health service utilization. The low acceptance rate of protective interventions (15%) reflects the powerful influence of gender role conflict theory, where traditional masculine norms (self-reliance, emotional stoicism, physical toughness) create cognitive dissonance when men experience victimization. The Theory of Planned Behavior (Reis et al., 2023) provides complementary theoretical insight into male victims’ decision-making regarding help-seeking. Behavioral intentions are shaped by three primary factors: attitudes toward the behavior, subjective norms, and perceived behavioral control. For male domestic violence victims, all three factors operate as barriers. Additionally, these behavioral barriers are further reinforced by institutional responses that may not recognize or validate male victimization, creating a self-perpetuating cycle of silence in which male victims avoid seeking help, services remain underdeveloped for male victims due to apparent low demand, and continued lack of appropriate services further discourages help-seeking.

From a clinical behavioral perspective, the predominance of minor injuries among male victims who do present to the ED suggests a possible reporting threshold effect. Men may only seek help when injuries are visible and undeniable, consistent with face-saving behaviors documented in masculinity research. The significant association between visible facial injuries and formal reporting (OR = 3.85), in fact, supports this interpretation and recent research from diverse cultural contexts reinforces these patterns. Qualitative studies from Kenya show that men prioritize informal supports and avoid formal reporting due to shame, fears about livelihood, and exaggerated masculinity expectations. Similarly, Japanese data indicate that physical violence (versus non-physical abuse) strongly predicts help-seeking, with men predominantly relying on informal forms of support, while non-physical abuse remains under-recognized (Stewart & Haselschwerdt, 2023, 2025; Kim et al., 2024; McLeod et al., 2024; Lane et al., 2023; Landa-Blanco & Mejía Sánchez, 2025). These cross-cultural patterns may suggest that core psychological and social mechanisms related to the role expectations of men operate across diverse settings to suppress male help-seeking for domestic violence.

Nevertheless, the study has some limitations to be considered. First of all, behavioral and psychological dimensions—such as fear, shame, or partner coercion—were not directly assessed and may have influenced both victim behavior and healthcare response. In other words, the existing data are insufficient to systematically improve understanding the behavioral frameworks explaining persistent under-reporting and the absence of qualitative data, such as victim narratives, also restricts deeper behavioral interpretation. Furthermore, the hospital-based sample represents only victims who sought emergency care, excluding those who never accessed services, likely the majority, given under-reporting estimates suggesting that 60–80% of male victims never disclose abuse. This selection bias limits generalizability to the broader male IPV victim population. Another limitation of this study consists of the incapability to systematically assess psychological outcomes (PTSD, depression, anxiety) or long-term behavioral consequences using validated instruments, which are critical for understanding the full impact of male victimization. Nevertheless, we lacked measures of injury severity (we used only the triage color code given by Health professionals in ED) and did not use IPV assessment instruments such as the Revised Conflict Tactics Scales (CTS2), limiting comparability with other studies. Moreover, cultural factors specific to the Italian context (e.g., family-centered values and specific gender role expectations) may affect help-seeking behaviors and may not generalize to other cultural settings. In conclusion, the relatively small sample size (n = 80) over six years may reflect both genuine low prevalence in ED presentations and the under-reporting of violence by men. This limited statistical power for subgroup analyses and precluded examination of potential effect modifiers.

Despite these limitations and given the scarcity of current literature addressing male domestic violence in healthcare settings, this investigation yields important practical implications for clinical practice, institutional policy, and future research directions. Healthcare systems should implement routine screening for DV and IPV using appropriate gender-neutral tools including modified versions of the Conflict Tactics Scale adapted for male victims, or the HITS instrument (Hurt, Insult, Threaten, Scream). The ED staff require comprehensive training to recognize subtle indicators of male victimization that may differ from typical female presentation patterns. Clinicians should maintain heightened awareness for inconsistent explanations for injuries, vague narratives that avoid identifying perpetrators or circumstances, or accounts that do not align with observed injury patterns. Defensive wound patterns merit particular attention. Repeated ED visits for “accidents,” falls, or unexplained injuries should be investigated and registered. Behavioral cues including anxiety, hypervigilance, reluctance to discuss injuries or circumstances, avoidance of eye contact, minimization of injury severity, or rapid affect changes when discussing the incident or relationship should be deepened. Clinical encounters with male DV victims should systematically incorporate evidence-based behavioral intervention strategies adapted for male-specific needs and barriers. Motivational interviewing techniques can effectively explore ambivalence about reporting and help-seeking without judgment, respecting patient autonomy and enhancing intrinsic motivation for change. Psychoeducation should explicitly normalize male victimization, directly challenging pervasive myths that domestic violence is exclusively a women’s issue or that men cannot be “real” victims, and providing concrete, specific information about available resources tailored to men. Individualized safety planning should be developed addressing realistic threats and practical strategies for protection. Connecting victims who have had similar experiences with peer support resources, e.g., male survivor support groups, can substantially reduce isolation. All interventions should be delivered within a comprehensive trauma-informed care framework that recognizes how traditional masculine socialization creates unique trauma responses and help-seeking barriers requiring adapted therapeutic approaches. At the institutional level, healthcare systems should undertake comprehensive reforms to ensure gender-inclusive domestic violence response establishing male-specific IPV protocols and resource materials with appropriate language, creating private, confidential spaces for disclosure separate from waiting areas, developing partnerships with men’s advocacy organizations and specialized services, implementing mandatory staff training on gender-inclusive IPV response and implicit bias, and monitoring and evaluating male victim outcomes systematically through quality improvement initiatives.

## 5. Conclusions

This study strengthens the scant literature systematically documenting male victims of domestic violence in an Italian emergency setting using standardized forensic and clinical protocols (Tuscany Rosa Code Protocol).

The investigation highlights that domestic violence against men, still under-recognized and under-reported, is a clinically relevant and socially important issue. Through a retrospective analysis of 80 male victims who accessed emergency services over a six-year period, we identified clear patterns of female-perpetrated intimate partner violence, frequent injuries to the upper body, and widespread reluctance to seek help or accept protective measures.

Although the physical injuries sustained were often classified as mild or not severe, the behavioral and psychological consequences may be far more serious and long-lasting. The low rate of legal reporting and refusal of social protection services suggest that male victims face unique sociocultural barriers—including stigma, gender norms, and institutional bias—that limit their access to care and justice. These findings underscore the urgent need to address systemic shortcomings in the recognition and support of male victims of domestic violence.

This study has several limitations that warrant consideration, and further studies are needed to overcome those boundaries. Perspective research should employ prospective designs with validated behavioral measures, qualitative interviews to explore lived experiences and decision-making processes, and longitudinal follow-up to assess help-seeking trajectories and intervention effectiveness. Mixed-methods approaches combining quantitative outcome data with in-depth qualitative exploration would provide richer understanding of the complex barriers male victims face.

Some recommendations deserve priority attention to move toward more equitable intervention models:

- Revise existing protocols (e.g., Rosa Code) to include male-specific risk factors, questions, and referral pathways;
- Train healthcare and forensic professionals to recognize non-obvious signs of male victimization and respond with gender-sensitive approaches;
- Improve data collection and central reporting systems to capture the prevalence, context, and outcomes of violence against men;
- Promote public health awareness campaigns that challenge stereotypes and encourage male help-seeking behavior.

## Figures and Tables

**Table 1:** Annual distribution and demographics of male victims.

	N	%
**Year**		
2017	25	31.2
2018	12	15
2019	13	16.3
2020	6	7.5
2021	8	10
2022	16	20
**Age of victims (years)**		
18–30	18	22.5
31–40	9	11.5
41–50	21	26.3
51–60	22	27.5
61–70	4	5
>70	6	7.5
**Nationality of victims**		
Italian	67	83.8
EU	4	5
Asiatic	1	1.2
African	6	7.5
South American	2	2.5

**Table 2:** Details about access to ED and perpetrators.

		N		%
ES accesses				
Time of access (h)	07:00 AM–01:59 PM	19		24.4
	02:00 PM–08:59 PM	36		46.2
	09:00 PM–06:59 AM	23		29.4
Timing between attack and ES access	0–1	8		10
	1–2	14		17.5
	2–8	14		17.5
	8–12	4		5
	12–24	9		11.3
	24–48	6		7.5
	More than 72 h	1		1.2
	NA *	24		30
Characteristics of perpetrators		M	F	
Current partner		2		2.5
			40	50
Former partner		-		-
			4	5
Parent		3		3.75
			1	1.25
Child		8		10
			2	2.5
Relative		6		7.5
			1	1.25
Other family members		5		6.25
			2	2.5
NA (6 cases)				7.5

* NA: not available.

**Table 3:** Injury characteristics.

	Site	n.	IPV	NON IPV	NA *
Abrasions		24	18	6	
	Head and neck	11	10	1	
	Head/neck, upper limbs/lower limbs, and trunk	13	8	5	
Contusion/bruises		17	9	7	1
	Head and neck	5	5		
	Upper limbs	5	2	3	
	Trunk	1		1	
	Lower limbs	1	1		
	More than one site				1
Lacerations	Head and neck	2		2	
Sharp wounds	Head/neck and upper limbs	1		1	
Burns	Upper limbs and trunk	1	1		
Skin redness	Lower limbs	1		1	
Functional impairment		5	2	3	
	Head and neck	1	1		
	Upper limbs	1	1		
	Trunk	1		1	
	NE	2		2	
Abrasions and contusions		3	2	1	
	Head and neck	1		1	
	Head/neck and upper limbs	2	2		
Abrasions and lacerations		2	1	1	
	More than one site (head/neck and upper limbs)	2	1	1	
	Head/neck and upper limbs and lower limbs				
Abrasions and contusions and lacerations	Head/neck and upper limbs and trunk	1		1	
Abrasions and bone fractures		2	1	1	
	Head and neck	1		1	
	Head/neck and upper limbs and lower limbs	1	1		
Abrasions and sharp wounds	Lower limbs and genital area	1			1
Abrasions and sharp wounds and functional impairment	Head/neck and trunk	1		1	
Abrasions and functional impairment	Upper limbs and lower limbs	1	1		
Contusions and lacerations		2		2	
	Head and neck	1		1	
	More than 2 sites	1		1	
Contusions and sharp wounds	Upper limbs and trunk	1	1		
Contusions and bone fractures	Head and neck	1	1		
Contusions and abrasions and functional impairment	Head/neck and upper limbs	1		1	
Contusions and lacerations and bone fractures		2	1	1	
	Head and neck	1	1		
	and other sites	1		1	
Contusions and lacerations and redness of skin	Head/neck and upper limbs	1	1		
Contusions and functional impairment	Head and neck	1		1	
Bites and contusions		2	2		
	More than one site	2	2		
Bites and lacerations	Upper limbs and trunk	1	1		
Bites and abrasions and lacerations		2	2		
	Head/neck and trunk	1	1		
	Head/neck and other sites	1	1		
Bites and contusions and abrasions	Head/neck and lower limbs	1	1		
Bites and contusions and functional impairment	Head/neck and upper limbs	1	1		
Only verbal		2		1	1

* NA: not available.

**Table 4:** Comparison of characteristics between reporters and non-reporters.

Characteristic	Reporters (n = 47)	Non-Reporters (n = 33)	*p*-Value
Age, mean (SD)	45.2 (14.3)	42.8 (16.1)	0.472
Italian nationality, n (%)	38 (80.9)	25 (75.8)	0.571
Female perpetrator, n (%)	41 (87.2)	15 (45.5)	<0.001
Visible facial injuries, n (%)	32 (68.1)	12 (35.3)	0.003
Multiple injury sites, n (%)	35 (74.5)	14 (42.4)	0.002
Accepted protective services, n (%)	12 (25.5)	0 (0.0)	0.001
Previous ED visits for DV *	16 (34)	4 (12.1)	0.012

* In our dataset, ‘previous ED visit for DV’ refers exclusively to physical violence, as no other forms of violence (economic, psychological, verbal, etc.) were reported.

**Table 5:** Reasons provided by the victims to the professional to not file formal reports and documented coping strategies.

Non-Reporting Reasons	Non-Reporters (n = 33)	% (of n = 33)
Shame and embarrassment about victimization	22	66.7%
Fear of not being believed by authorities	15	45.5%
Fear of perpetrator retaliation or escalation	11	33.3%
Desire to protect children from family disruption	8	24.2%
Belief that violence would stop without intervention	7	21.2%
Previous negative experience with legal/social services	6	18.2%
Concern about loss of custody or parental rights	5	15.2%
**Adopted coping strategies**		
Denial or minimization of abuse severity	31	38.8%
Avoidance of perpetrator or separation	28	35%
Seeking informal support from friends/family	24	30%
Social withdrawal and isolation	18	22.5%
Substance use (alcohol/drugs) to manage distress	12	15%

## Data Availability

The original contributions presented in this study are included in the article. Further inquiries can be directed to the corresponding author.
